# Complementarity of Matrix- and Nanostructure-Assisted Laser Desorption/Ionization Approaches

**DOI:** 10.3390/nano9020260

**Published:** 2019-02-14

**Authors:** Pawel Pomastowski, Boguslaw Buszewski

**Affiliations:** 1 Interdisciplinary Center for Modern Technologies, Nicolaus Copernicus University in Torun, Wilenska 4, 87-100 Torun, Poland; bbusz@umk.pl; 2 Department of Environmental Chemistry and Bioanalytics, Faculty of Chemistry Nicolaus Copernicus University in Torun, Gagarin 7, 87-100 Torun, Poland

**Keywords:** matrix-assisted laser desorption/ionization, nanostructures-assisted laser desorption/ionization, MALDI, NALDI, bioanalytics, nanoparticles

## Abstract

In recent years, matrix-assisted laser desorption/ionization (MALDI) has become the main tool for the study of biological macromolecules, such as protein nano-machines, especially in the determination of their molecular masses, structure, and post-translational modifications. A key role in the classical process of desorption and ionization of the sample is played by a matrix, usually a low-molecular weight weak organic acid. Unfortunately, the interpretation of mass spectra in the mass range of below *m/z* 500 is difficult, and hence the analysis of low molecular weight compounds in a matrix-assisted system is an analytical challenge. Replacing the classical matrix with nanomaterials, e.g., silver nanoparticles, allows improvement of the selectivity and sensitivity of spectrometric measurement of biologically important small molecules. Nowadays, the nanostructure-assisted laser desorption/ionization (NALDI) approach complements the classic MALDI in the field of modern bioanalytics. In particular, the aim of this work is to review the recent advances in MALDI and NALDI approaches.

## 1. Introduction

The problematic nature of modern bioanalytical research is mainly related to the complex biological matrix and the extremely low concentration of tested analytes in the sample, the amount of which is usually small and often representative for several cells (e.g., cellular lysates). The consequence of the above-mentioned features of biological materials is the need to look for highly effective, efficient, and sensitive analytical methods that allow for detection of, on the one hand, macromolecules, and on the other hand, low molecular compounds. These requirements can be met by spectrometric techniques. Mass spectrometry (MS), besides spectroscopic techniques, e.g. nuclear magnetic resonance (NMR), Fourier-transform infrared spectroscopy (FTIR), ultraviolet–visible (UV-VIS) spectroscopy, or fluorescence based-methods, is the basic approach in identification of biologically active compounds. Its advantage is it has very high sensitivity, which in combination with other analytical techniques, such as liquid chromatography (LC-MS) or electrophoretic (e.g., capillary zone electrophoresis (CZE-MS) and two-dimensional gel electrophoresis (2D-GE)) approach, gives a powerful tool for analysis of very complex mixtures, e.g., peptides and proteins, such as casein fractions [1], or even proteins of microbial cells [2]. Despite the huge variety of mass spectrometry techniques, the construction design in all the instruments is similar. The main elements are the source of ions (which ionizes the molecules), the analyzer (in which the separation of ions occurs due to the mass-to-charge (*m/z*) ratio), the detector, and the recorder [3]. 

The aim of this work is to review the recent advances in matrix-assisted laser desorption/ionization (MALDI) and nanostructure-assisted laser desorption/ionization (NALDI) approaches, especially utilization of metal and metal oxide nanoparticles for laser desorption/ionization (LID) approaches. 

## 2. Matrix-Assisted Laser Desorption/Ionization

**Figure 1:**
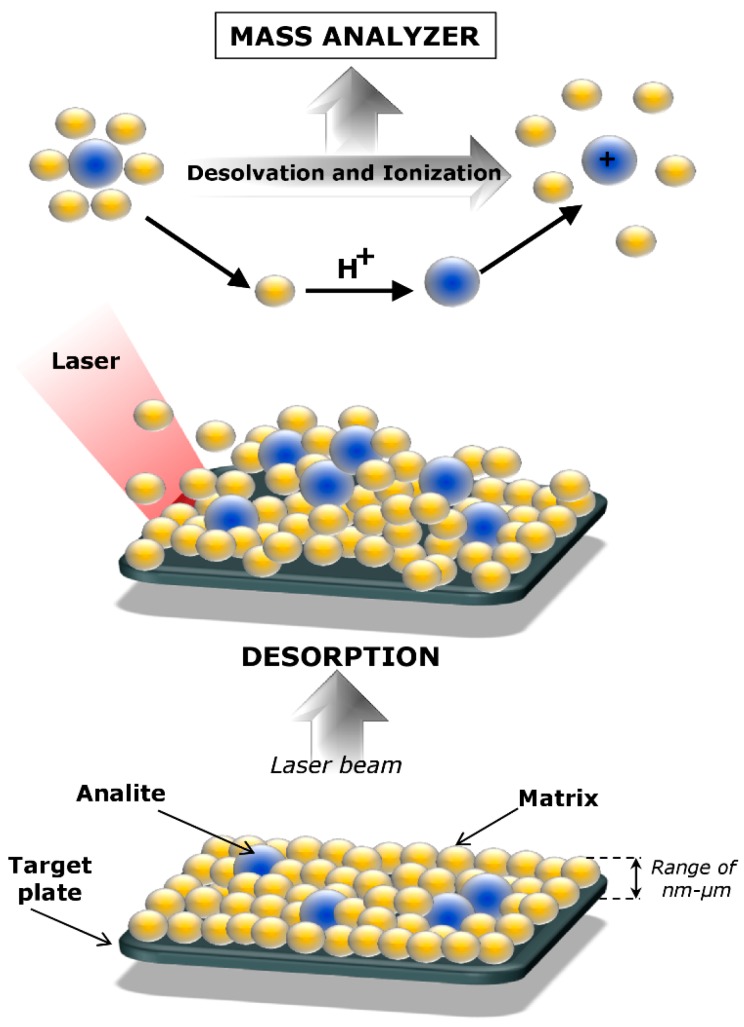
Ionization and desorption processes in matrix-assisted laser desorption/ionization (MALDI).

Matrix-assisted laser desorption/ionization is currently used in mass spectrometry techniques to generate ions by using a laser mostly in UV range. MALDI is an ionization technique developed in mass spectrometry since the mid-1980s, most often in combination with a time-of-flight (TOF) analyzer, operating mainly under high vacuum conditions, but also under atmospheric pressure [4]. It allows an analysis of labile molecules in the gas phase in the form of pseudo-molecular ions. Hence MALDI, as well as electrospray (ESI), belongs to the group of soft ionization techniques. On the other hand, the advantage of MALDI compared to the ESI ionization technique is its greater tolerance for the presence of salts or detergents in the samples in the case of analysis of proteins isolated from various biological matrices and purified by electrophoretic techniques, e.g., two-dimensional gel electrophoresis (2D GE) [5]. Ionization in the MALDI is the result of laser radiation on a crystallized-mixture of the sample and matrix. The matrix mediates the transfer of energy to the test substance, and facilitates ionization and desorption of the sample. The purpose of the matrix is to ionize the molecules, which cannot absorb laser radiation. Therefore, ionization proceeds indirectly (Figure 1) [6].

The matrices used in the MALDI technique are substances that absorb UV radiation well, easily sublimate, and after desorption supply large amounts of ions (protons) needed for the ionization of the analyte [7]. The most commonly used matrices in MALDI are α-cyano-4-hydroxycinnamic acids (HCCA, CHCA) enabling the analysis of proteins, peptides, and lipids mostly in the bottom up approach by application of classical digestion by trypsin in-solution or immobilized enzyme microreactors (μ-IMER) used for transferrin and bovine serum album analysis, respectively [8,9].

For the top down strategy, sinapic acid (SA), enabling the analysis of proteins and peptides > 3 kDa, is most commonly used. However, 2,5-dihydroxy acid benzoic (DHB) allows the analysis of phospholipids, proteins and peptides > 3 kDa, polar polymers, and lipids, while ditranol (DIT) makes it possible to detect nonpolar polymers and glycolipids [10]. The 1,5-Diaminonaphthalene (1,5-DAN) is used for de novo sequencing of peptides [5,11]. In addition to choosing a matrix, the method of applying the sample to the target also has a large impact on the success of the analysis. The method most commonly used for applying matrices to a MALDI target is called dry droplet method, where drops of the matrix solution and samples are mixed together before being applied to the plate. This method has been described, among others, in the analysis of disulfide-bonded peptides with using a mixed matrix of 2-(4-hydroxyphenylazo)benzoic acid and α-cyano-4-hydroxycinnamic acid [12]. The advantages of the method are the stability of the samples for a long time, and the possibility of washing the precipitated crystals with water to get rid of the salts [12]. Additionally, the method of evaporation under a vacuum can be used, where after applying a drop on the plate, the solvent is immediately evaporated in a vacuum, allowing for more homogeneous distribution, e.g., in the case of analysis of lipid mixtures isolated from mouse brain tissue [13]. In the thin layer method (e.g., three layer, sandwich methods), the matrix and sample are applied separately in a fast evaporating solvent, and it is then applied to the sample. High homogeneity of the matrix layer obtained in this way enables high repeatability of the analysis. The three-layer method for protein analysis at low concentration contaminated by salts and buffers [14], or the sandwich method [15] used for lysates of single mammalian cell analysis were applied. 

During MALDI ionization, positive and negative ions may be formed. In the case of biological samples, various types of [M+H]^+^, [M+Na]^+^, and [M+K]^+^ adducts are formed. On the other hand, polymers such as polysterene primarily produce ions stabilized with metal cations (predominantly sodium) [16]. Ions containing a matrix molecule, or a particle formed as a result of its decay, are generally formed in a low yield. For the stabilization of ions obtained from DIT, the addition of silver cations [M+Ag]^+^ is very often used [17]. In other cases, under the influence of high temperature, the subtraction of water from the molecule may occur [M-H_2_O+H]^+^, especially during MALDI imaging of lipids in different tissues [18]. The most frequent signals in the spectra are the intense molecular peak type [M+H]^+^ and a small number of multiply charged ions of [M+nH]^n+^ type and [nM+H]^+^ type. The last two types of ions are formed more often by the ionization of peptides and molecules with a large surface area. Occasionally, real molecular ions of the [M]^+^ type are formed in the MALDI technique [19].

MALDI is one of the soft methods of ionization. Added in excess, the matrix separates the molecules of the test substance from itself, and without fragmentation, transfers the energy absorbed by them. Because the main part of heat energy is transformed into the energy of vibrations of chemical bonds, absorbed by the matrix molecules, it protects the test substance against degradation [20]. On the other hand, the ions of low molecular weight are often fragmented under these conditions. This paradox is explained by the fact that large particles have many possibilities of vibration and rotation, so that the absorbed energy is dispersed without breaking the chemical bonds [19,20]. 

In MALDI, the phenomenon of fragmentation (of both proteins and polypeptides) is practically non-existent, and, as a result, no signals from fragmentation ions (resulting from the disintegration of particles in the ionizer) are observed in the spectra. In this situation, signals from the matrix or its adducts with the components of the analyzed sample (e.g. adduct [MH]^+^) are presented. A case requiring special attention is the so-called cluster (matrix-cluster), i.e., a combination of several ions that are part of the matrix-giving signals on the spectrum, significantly impeding the interpretation of the obtained results [21]. Although the intensity of the signals coming from the matrix decreases with increasing mass, in the case of a small amount of digested protein (in the order of picomoles), they may interfere with signals originating from polypeptide fragments in the mass range of up to approximately 2 kDa. Molecules of CHCA matrix and K^+^, Na^+^, and H^+^ ions form clusters [21,22]. Complete elimination of both sodium and potassium ions from the system (e.g., used solution) is practically impossible (for example due to the use of laboratory glassware), therefore on MALDI spectra also CHCA matrix-cluster peaks are present. These complexes (clusters) are formed by interaction of the cations of the discussed metals with the carboxyl group or π electrons of the aromatic ring of the CHCA molecule (so-called sandwich structures), and depending on the number of molecules involved in the cluster, they can take various masses. Matrix clusters are formed only and exclusively in the presence of organic compounds—during the co-crystallization of CHCA with the analyte, causing suppression and difficulties in the analysis of low-molecular compounds [22].

## 3. Protein Identification and Analysis by MALDI-TOF Mass Spectrometry

Proteins are a large and important group of biomolecules that play an extremely crucial role in cells and tissues. Therefore, the identification and analysis of proteins have become a key point not only in biochemistry, but also in analytical chemistry, medicine, and industry. There are a lot of well-known methods commonly used for protein separation (e.g., SDS-PAGE, isoelectric focusing, or two-dimensional gel electrophoresis), detection of specific proteins in a sample (immunoblotting), and for protein identification (such as Edman degradation and mass spectrometry) [5,23,24,25]. Since the late 1980s, the mass spectrometry technology, especially introducing of matrix-assisted laser desorption/ionization time-of-flight mass spectrometry (MALDI-TOF MS), has been applied for protein analysis [4], and has become one of the fundamental approaches used for this purpose—mainly due to its simplicity, sensitivity, and large mass range. Moreover, the method is relatively resistant to interference with matrices commonly used in protein chemistry [26]. In cells, proteins can undergo a wide variety of post-translational modifications (PTMs), which strongly affect their functionality, structure, or activity. Mass spectrometry currently provides one of the best and most effective methods for investigating and locating PTMs. One example can be phosphorylation, which is often identified after enzymatic digestion of a phosphoprotein by either MALDI-TOF MS or electrospray mass spectrometry [27]. Locke et al. [28] have identified the PTM of connexin26 (Cx26) by using a MALDI-TOF MS approach. Mutations in the Cx26 gene are the main cause of inherited non-syndromic deafness, therefore the application of mass spectrometry allowed identification and determination of whether post-translation modification of the studied protein occurs at sites of the disease-causing mutations. According to the data from this study, several presumed PTMs of connexin26 were identified, such as acetylation, hydroxylation, γ-carboxyglutamation, methylation, and phosphorylation [28]. Additionally, analysis have also confirmed that many of the identified PTMs sites are also present at sites of Cx26 disease-causing mutation. MALDI-TOF MS has also been widely used for determination of post-translation modification of milk proteins, such as immunoglobulins [29], α-lactalbumin [29,30,31,32], κ-casein [1,33], and lactoferrin [34,35,36]. Pomastowski et al. [1] have carried out the separation of the bovine milk casein (α-, β- and κ-casein) components and used a MALDI-TOF-MS method for their detailed identification and characterization. The separation of milk casein fractions was performed using HPLC gradient elution and after the chromatographic separation, the intact protein analysis for the obtained samples was applied. It was possible to register MS spectra for the three types of casein component, α_s1_-CN, β-CN, and κ-CN, respectively. Pomastowski with co-workers [1] have also subjected the MS/MS spectra of the obtained casein peptides to the analysis of phosphorylation sites—the obtained results have pointed out the presence of the PTMs in the sequences belonging to all the separated casein fractions. In the work of Ham et al. [32], the determination of caprine milk major proteins’ molecular weights (MW) was performed by using MALDI-TOF MS method. Due to the problem of the decreased intensity of casein mass signals with the TFA reagent, which is commonly used in the MALDI technique, Ham and colleagues [32] have proposed an alternative and improved a sample preparation method—the use of capillary electrophoresis reduction buffer before the TFA dilution. Based on the received data, the molecular mass of α-lactalbumin, β-lactoglobulin, and α- and β-casein were found to be at the level of 14, 18, 23, and 23 kDa, respectively. In comparison with cow (bovine) milk, the MW of β-lactoglobulin in caprine milk was higher, whereas the mass of β-casein was lower. What is more, the used protocol has shown that CN mass signals can be enhanced by a CE reduction buffer treatment. One of the most-studied PTMs is glycosylation—over the past decade, many research groups have been working on the qualitative and quantitative measurement and monitoring of glycans attached to proteins [29,30,37,38,39]. Recently, a study on proteins of early lactation bovine milk using MALDI-TOF/TOF MS approach to monitor changes in time of N-glycan classes was reported [29]. The samples were collected in specific time intervals (1 day; 1, 2, 3, and 4 weeks postpartum). The results of this work have shown that the glycomic profile of colostrum on day 1 after calving was fundamentally different than that for samples collected in other periods during early lactation. The proteins detected in colostrum samples from the first day postpartum were more highly sialylated than milk samples obtained at other time points. Moreover, the ratio of glycolylneuraminic acid (Neu5Gc) to N-acetylneuraminic acid (Neu5Ac) was also significantly higher on day 1 and decreased gradually with time. Quaranta with co-workers [39] have examined the N-glycan profile of transferrin by the use of the microfluidic CD platform for selective capture of protein from human serum samples and for preparation of its N-linked glycans for MALDI-MS method. Data from this study indicated that the non-fucosylated biantennary glycan (*m/z* 1664) was the most abundant glycan in all the analyzed samples. Another approach is described in the work of Yang et al. [33], who have applied the two-dimensional gels coupled with MALDI-TOF MS for detection of cow milk adulteration in different types of milk mixtures (buffalo, yak, camel). The distributions of protein spots of α_s1_-casein, α-lactalbumin, and β-lactoglobulin on gel maps were used for this purpose—especially β-lactoglobulin from cow, goat, yak, and buffalo milk, and α-lactalbumin from camel milk allowed for detection of the adulteration at the 0.5% level. 

Nowadays, designing new treatments and drugs generates much more interest—creating new medicines or bioactive complexes requires a development of the procedure which will allow for effective identification and characterization of the obtained products. Matrix-assisted laser desorption ionization–time of flight (MALDI-TOF) mass spectrometry seems to meet all the required criteria. Žuvela et al. [40] have examined the quantitative structure–drug–property relationships and molecular simulation of the carbonic anhydrase IX-sulphonamide complexes. The use of MALDI-TOF/TOF MS, as a great supplement of the conducted simulations, allowed for the determination of CA IX and CA IX–inhibitor complexes molecular mass (Table 1) and establishment of their formation. The registered MALDI spectra confirmed the binding of one molecule of inhibitor C75 and two molecules of C84 inhibitor to the CA IX protein [40]. This data clearly highlights that mass spectrometry may play an important role in the development of new drugs by the characterization of interactions between the enzymes and inhibitors [40,41]. Another work described by Liu et al. [36] is based on the determination of specific complexes of chlorogenic acid (CA) and lactofferin (LTF). Such a protein conjugation was proposed as an effective approach to overcome peptides’ instability under unfavorable conditions (e.g., organic solvents, heating), and accordingly, to increase their use in the food industry. Similarly to the previous paper, matrix-assisted laser desorption/ionization time-of-flight mass spectrometry was applied to confirm the formation of the covalent glycoslated CA–LTF conjugates; data from this study provided the mass of native LTF, shown as a *m/z* 84011.15. In the case of the proposed chlorogenic acid-lactoferrin conjugate, Liu with co-workers [35] have observed an increase in the molecular weight associated with the three CA molecules bound to one protein molecule. 

Recently, multifunctional lactoferrin belonging to the transferrin family has been raising interest in many scientific and industry fields, mainly due to its health benefits, biocompatibility, and antimicrobial activity against a wide spectrum of microorganisms [42,43,44]. Pomastowski et al. [34] have used lactoferrin as a good template for the immobilization of silver ions into protein, and furthermore, to determine the antibacterial activity of the received Ag-LTF nanocomplexes. In the mentioned work, the MALDI-TOF/TOF MS approach was used for the identification and characterization of lactoferrin isolated from bovine whey. As matrices, the α-cyano-4-hydroxycinnamic and sinapinic acids were used; before the analysis, the protein underwent trypsin digestion. Intact MALDI-TOF MS analysis allowed for precise determination of the LTF mass, which was marked in the range of 77.167–81.189 kDa. Moreover, the registered differences in the masses are related to glycosylation of lactoferrin. The sequence coverage for the described protein was 19–25%. 

Meller et al. have designed the capillary-based microreactors with immobilized enzymes, such as trypsin and chymotrypsin [8,9], as well as the evaluation of its usefulness in proteomic research. The work from 2016 described the investigation of a process using trypsin and chymotrypsin immobilized on the μ-IMER reactor against human transferrin as a substrate, and the hydrolysis products were analyzed with MALDI-TOF. The application of the described microreactor have resulted in transferrin sequence coverage in the range of 41.8–56.4%—it was significantly higher than the SC obtained for the classical protocol, including in-solution digestion (only 17.8%) [9]. Similarly, the paper from 2017 [8] is based on the examination of the activity of two prepared microreactors (TS1 and TS2) using bovine serum albumin (BSA). Data from the BSA digestion collected during MALDI-TOF MS analysis have shown the sequence coverage of 43.9% and 35.7% in the case of TS1 and T1 microreactors, respectively [8]. All of the designed microreactors can be used as a model system imitating the intestinal digestion of proteins or even as an efficient tool for studying the protein biotransformation. Importantly, MALDI-TOF MS plays a crucial role as a complementary technique needed for the evaluation and characterization of the obtained system. Details of the described studies are summarized in Table 1. 

### Problems with Protein Identification and Analysis by MALDI-TOF Mass Spectrometry

MALDI-TOF MS has become an important analytical tool for identifying and analyzing proteome and it brings advantages such as high detection sensitivity or fully automated analysis of many samples in a short time. Although the MALDI approach is very powerful, such a technique often requires specific sample preparation steps which can create artifacts—it is frequently observed that it has a major effect on the outcome of the recorded ion signal. At the sample preparation level, parameters such as relative humidity, temperature, air flow, presence of a buffer, salts, or a target surface can considerably influence a MALDI sample; an incorrect sample preparation process can result in matrix/analyte co-crystallization, which will strongly affect the desorption and ion formation processes [47]. Another critical step in the preparation of proteins for analysis with mass spectrometry is proteolysis [48]. Mainly due to the fact that trypsin digestion is believed to be predictable and inexpensive, it is the preferred reagent in most digestion protocols [49]. Results of tryptic digestion are peptides that contain a basic residue at the C-terminus, making the obtained spectra of peptides relatively easy to interpret. Despite its advantages, this approach can also affect the proper identification or analysis of protein by MALDI. One of the drawbacks can be the rate of protein digestion, which demands significant limitations on high-throughput peptide identification. The solution of this problem can be an approach proposed by Russel et al. [48]. They have proved that digestion of proteins by using mixed-solvent systems (methanol-water, acetonitrile-water, 2-propanol-water, or acetone-water) for the MALDI-TOF MS purpose is much more effective than traditional methods. The problem can be also uncontrolled hydrolysis of the prepared peptides on the MALDI matrix [49]. The influence of matrix solution and its preparation conditions also play a crucial role in the MALDI-MS analysis of peptides and proteins [49,50]. Cohen and Chait [51] have performed a study based on the determination of the MALDI matrix type on the mass discrimination effects. Their results indicated that the discrimination effects are strongly determined by the composition of sample−matrix solution, the rates at which the sample–matrix co-crystals are grown, and pH of the reaction [52]. There are a lot of reports describing the application of MALDI-TOF MS, but there are still many challenges fundamental to its ionization process. These limitations are mainly related to factors such as the sample–matrix heterogeneity or the limited dynamic range due to detector saturation. As a consequence, these combined factors are thought to compromise the accuracy, precision, and utility [53]. Another challenge in protein determination by MALDI-TOF MS is the reproducibility of peak intensity [54]. The main goal of MALDI protein profiling is to identify differences in peak quality, as well as differences in peak intensities between the investigated sample and the control one. Therefore, the reproducibility of this parameter seems to be of highest importance. Unfortunately, the poor reproducibility was found to be one of the main problems during protein analysis by MALDI approach. One of the reasons can be the matrix (co)crystallization—Cohen and Chait [51], as well as Amado et al. [54], have indicated the processes occurring during the sample and matrix preparation. Different matrix molecules may crystallize in different shapes and dimensions; additionally, proteins are likely to accumulate at the droplet periphery, so the composition of the matrix solution and the rate of crystal growth strongly influence the spectral output [53,54]. Kratzer et al. [55] have described the ion-suppression effect which occurs when an ion suppresses the peak signal of other ions in the sample, and peptides with greater hydrophobicity show the greatest suppression effects. Furthermore, one of studies performed by Juhasz and Biemann [56] found that highly acidic compounds are responsible for producing weak signals in MALDI-TOF MS, but the mixing of them with a basic peptide and forming noncovalent complexes caused the signals to improve.

## 4. Non-Protein Analysis by MALDI-TOF Mass Spectrometry

The classical MALDI-TOF MS technique is also widely used for lower molecular compounds determination (up to 2000 Da). Al-Soud et al. [57] used this technique for the identification of cyclitols derived from different parts of *Medicago sativa*. For extract preparation and purification, the Soxhlet extraction and solid-phase extraction were used. After that, portions of each extract solution (in 1% TFA) were mixed with α-cyano-4-hyroxycinnamic acid (HCCA) matrix at the ratio of 1:1 and analyzed using MALDI-TOF MS. The obtained results allowed for identification of three cyclitols (D-chiro-inositol, D-pinitol, and L-chiro-inositol) in different parts of *Medicago sativa* (stem, flowers, and leaves). MALDI technique proved to be a sensitive tool for the rapid identification and differentiation of cyclitols enantiomers in plant extracts. In turn, Ricci at al. [58] profiled the food-grade plant extracts due to the content of tannins, using MALDI-TOF MS and UV-vis spectrophotometry. Picariello et al. [59] comparatively semi-quantified and characterized flavonoids from Prosopis nigra, Prosopis alba, Prosopis ruscifolia germ, and one European carob species by off-line coupled RP-HPLC and MALDI-TOF MS. Schaftoside and isoschaftoside had a dominant share of phenolic compounds. This technique was also used for characterization of Acacia mangium polyflavonoid tannin by Hoong et al. [60]. They obtained a series of signals derived from condensed tannins oligomers of up to 11 flavonoid units. The results indicate that condensed tannins of A. mangium consists mainly of prorobinetinidin in combination with prodelphinidin and profisetinidin. The evaluation of these compounds by other methods is quite difficult and the obtained results provide that MALDI can be a useful method for easy determination of polymer chain length distribution, frequency of monomer unit, and degree of polymerisation. Sastre et al. [61] developed a procedure for *Yucca schidigera* saponins determination based on the of intact saponins sodium adducts analysis, as well as the oligosaccharidic chain ions using MALDI-TOF MS technique. 

Szultka-Mlynska et al. [62] proposed the use of classical MALDI technique coupled off-line with HPLC for identification and determination of antibiotics and their metabolites in human blood and tissue after a single oral dose. They used three matrices: HCCA, DHB, and sDHB (9:1 mixture of DHB and 2-hydroxy-5-methoxybenzoic acid). DHB and sDHB proved to be the best matrices in the analysis of antibiotics. Application of these matrices ensured the stable ionization, high reproducibility, and resolution of registered spectra. The research allowed for determination of the structures of potential antibiotic metabolites based on the measurement of exact mass product ions. Therefore, the MALDI technique can be used in the analysis of real samples in clinical laboratories as a tool for the monitoring antibiotics during therapy. Amelin et al. [63] proposed an analytical method for the determination and identification of residual amounts of antibiotic drugs in food and feeds. In their experiment, they compared the classical MALDI and Surface-assisted laser desorption/ionization (SALDI)-MS with graphite matrix method. The 26 antibiotics were investigated and the obtained limits of detection was 0.01–0.3 μg/kg and 0.001–0.03 μg/kg for MALDI and SALDI-MS, respectively. Grant et al. [64] used the combination of the three-stage solid phase immunoextraction (SPIE) system and MALDI technique for determination of trace-level of sulfamethazine in complex environmental samples. The developed method proved to be effective and the analysis time did not exceed two hours. 

Jeyakumar et al. [65] applied classical MALDI to develop a highly sensitive, reliable, rapid, and simple method for mycotoxin detection in Fusarium species-infected sugarcane. The proposed methodology led to determination of six mycotoxins: fumonisin B1, fumonisin B2, nivalenol, zearalenone, aflatoxin G1, and 3-acetyl-deoxynivalenol. MALDI-TOF was also used for determination of lipids by Walczak et al. [10]. In their experiment, polar lipids from milk were separated using high performed liquid chromatography (HPLC) and then identified by MALDI. According to the authors, the use of such a combination could be an alternative for the LC-ESI MS technique, which in the analysis of lipids encounters many problems, such as difficult interpretation of data, sensitivity to salinity of the sample, and lower sensitivity than MALDI. Using the proposed approach, they quantified six classes of phospholipids in powdered and raw milk extracts. Moreover, it allowed for comparison of the fatty acid composition in powdered and raw milk. In the tested samples, the combinations of 46 fatty acids were identified, as well as 21 and 34 molecular species for powdered and raw milk, respectively. Research conducted by Schiller et al. [66] has shown that MALDI-TOF MS technique provides the information about fatty acid composition of human lipoproteins. Serna et al. [67] performed quantitative analysis of plasma lipids by MALDI-TOF MS in combination with HPLC-ELSD. They confirmed that the MALDI technique is a suitable tool for studying the linearity and repeatability for phosphatidylethanolamine (PE), triglycerides (TG), and phosphatidylcholine (PC). Hirata et al. [68] used MALDI-TOF MS technique to confirm the synthesis of glycolipids composed of lipids, glycerol, and rare sugars. Adeuya et al. [69] used this analytical method for determination of stereochemical arrangement and the number of hydroxyl groups in carbohydrates. In the first step, they applied vinyl acetate in order to selectively derivatize hydroxyl groups of the tested compounds and then recorded the MALDI mass spectrum. The obtained results indicate that this approach is a rapid aqueous-based method for enumeration of the carbohydrates hydroxyl groups and is applicable to single as well as multi-component mixtures. Abe et al. [70] determined the structural conformation of 11 oligosaccharides isolated from sugar beet molasses. The polymerization degree of the tested saccharides was defined as three by measurement of [M+Na]^+^ ions. Longuespée et al. [71] used MALDI for determination of the D-2-hydroksyglutarate (D-2HG) in brain tumor tissues. The quantity of D-2HG in tumor tissues increases as a result of isocitrate dehydrogenase genes mutations (IDH). These mutations have been detected in many tumor types. In the conducted experiments, 26 tumor tissues with known IDH mutations were analyzed and then the results were compared with these from samples with wild type IDH status. For all of the tested IDH mutant tissues, the clear signal derived from 2HG was observed, while for any of IDH wild type samples, it did not appear. Moreover, the detection of 2HG by MALDI-TOF MS system lasted less than 5 min. 

MALDI-TOF method has also found application in the analysis and characteristics of polymers. Yang et al. [72] tested different matrices for the analysis of Polyhexamethylene biguanide (PHMB), Poly(hexamethylene guanidine) chloride (PHMG), and Poly-[2-(2-ethoxy)-ethoxyethyl]-guanidinium-chloride (PEEG). They have found that there is no unique matrix which can ensure appropriate conditions of analysis for all the tested guanidines, due to the presence of extra C-O bonds in PEEG. The obtained results indicated that for PHMB and PHMG, the best matrix was HCCA and SA, while for PEEG, the best results were noted for DHB and 2-(4-Hydroxybenzeneazo)benzoic acid (HABA). In turn, Wu et al. [73] investigated the effect of solvents on polymer analysis by MALDI technique. As a model polymer they chose nylon-6. The solvents for nylon-6 were formic acid (FA) and trifluoethanol (TFE), while the solvents for HABA matrix were tetrahydrofuran (THF), formic acid (FA), and trifluoethanol (TFE). In this way, six different conditions of analysis were obtained. The results indicate that solvents have a significant effect on the analysis of synthetic polymers by MALDI. Moreover, the incorporation between matrix and analyte is meaningful in such analysis. Li et al. [74] used MALDI-TOF MS for characterization of polymers containing an azide group. Wetzel et al. [75] examined the effect of different parameters on the analysis of synthetic polymers by MALDI-TOF MS technique, such as laser energy, detector voltage, extraction voltage, delay time, lens voltage, matrix, and polymer concentration. The results have proved that the biggest impacts on the quality of the spectrum for polystyrene were the delay time and detector voltage when all-trans-retinoic acid (RA) were used as a matrix. In the case when dithranol was used as the matrix, three parameters were the most important: laser energy, delay time, and detector voltage.

MALDI technique is also useful for the analysis of nanocomposites of a metallic core and an organic deposit, as was shown by Railean-Plugaru et al. [76] research. They proved that MALDI-TOF/TOF mass spectrometry is a complementary tool for the determination of amino acids in organic deposits, as well as for recording silver clusters. Their further research [77] was aimed at deep characterization of silver nanocomposites fractions obtained after its field-flow-field fractionation (4F), as well as at comparison with native samples. It allowed for monitoring of changes resulting from the use of various buffers at organic deposits on the surface of metal core. The obtained results indicate that the tested particles are characterized by an uncommon cluster structure and can be described as a composition of two or more clusters of silver. Furthermore, one- and two-dimensional MALDI MS allowed for recording of the molecular fingerprint spectrum of the analyzed sample and examination of the structure of the parent ion, respectively.

## 5. Nanostructure-Assisted Laser Desorption/Ionization

Despite the large amount of research, detection of low molecular weight (LMW) compounds by MALDI-MS is a current analytical challenge. Application of MALDI for analysis of low molecular weight compounds (<700 Da) causes signal supersession. It is caused by high chemical background in the low mass region (up to *m/z* 800), which adversely affects sensitivity. In addition, acidity of matrix solution is problematic for small acid-labile bioactive compounds [78]. To resolve the problem of matrix presence, the desorption–ionization on porous silicon (DIOS) was introduced [79]. DIOS allows direct analysis of low molecular weight compounds (sugars, peptides, glycolipids, drugs [79]) using plates made of porous silicon DIOS or porous silicon dioxide (DIOSD). In the case of the DIOS/DIOSD technique, the matrix function is performed by porous silicon. The working principle of this technique consists of trapping analytes deposited on the surface, followed by laser irradiation and ionization [79]. Moreover, DIOS for detection of methamphetamine (MA), 3,4-methylenedioxymethamphetamine (MDMA), and delta-9-tetrahydrocannabinol (THC) in saliva [80], catecholamines [81], nucleotide, and oligopeptides [82], was applied.

**Figure 2:**
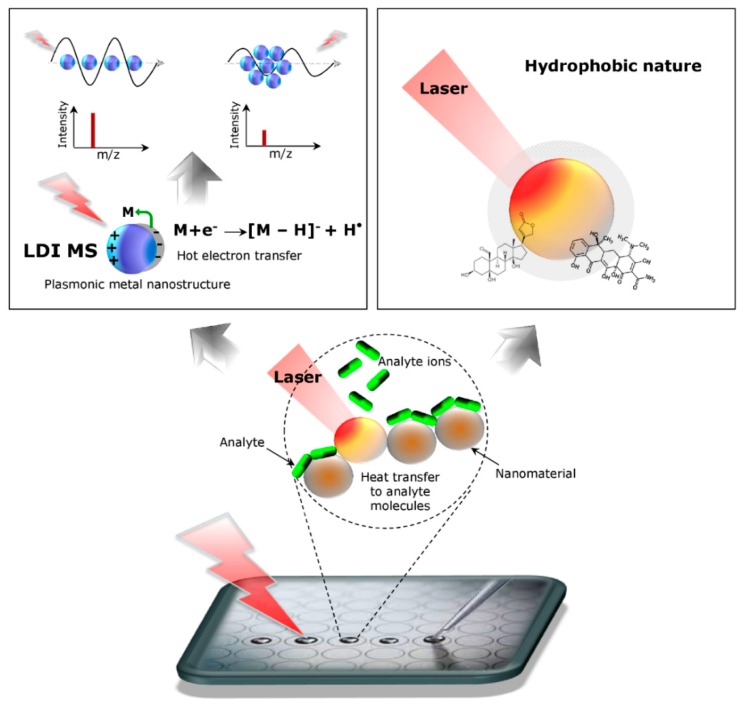
Mechanism of ionization in nanostructure-assisted laser desorption/ionization (NALDI).

Actually, organic matrix molecules can be also replaced by nanomaterials to assist ionization. It represents the field of nano-assisted laser desorption ionization (NALDI) called a nanostructure- and nanoparticle assisted laser desorption/ionization mass spectrometry (NPs-ALDI-MS) [83]. Abdelhamidhe reported that mechanism ionization in NALDI system is still not clear, and is “ill-defined” [84]. However, the main theory of LID in NALDI is explained by surface plasmon resonance (SPR). Nanoparticle systems (e.g., AuNPs) are a donor of charge, as the laser causes hot transfer of electrons from NPs to analyte molecules as a consequence of the ionization [83] (Figure 2).

Moreover, low molecular weight compounds in NALDI system of tungsten oxide and rhenium oxide particles showed a significant increase in the sensitivity of the measurement compared to the classical MALDI type analysis. It is caused by the strongly hydrophobic surface of the plate in the NALDI system (Figure 2). This property is used to purify the sample or even to isolate interesting compounds from more complex mixtures (e.g., blood, serum) analogously to solid phase microextraction procedure (SPME) [62]. After applying the sample to the surface of the plate, the hydrophobic compounds are adsorbed. After washing the surface, substances that may interfere with the measurement (interferents), such as salts and other components of the sample, are removed. The remaining analytes (e.g., metabolites of drugs) are selectively enriched with low matrix inference and high reproducibility [85].

**Figure 3:**
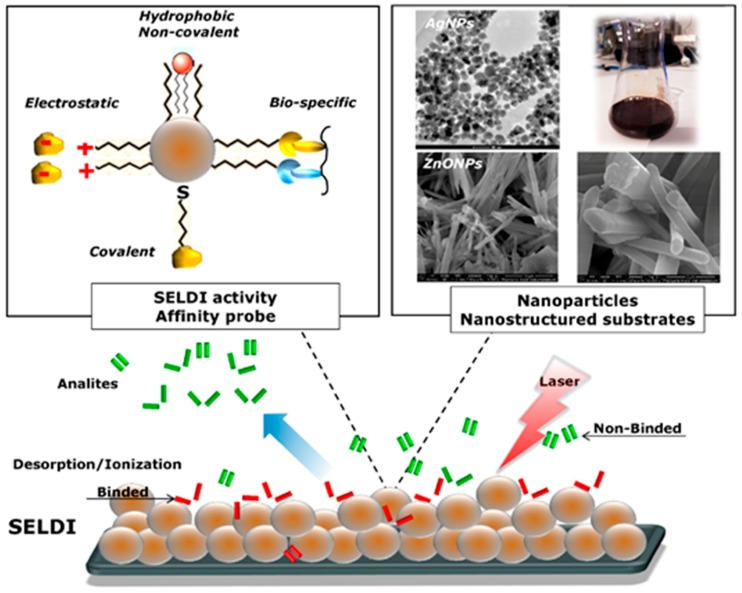
Principle of surface-enhanced laser desorption/ionization approach.

A special type of selective enrichment of the sample through adsorption of a specific group of substances based on nanomaterials is surface-enhanced laser desorption/ionization (SELDI). Surface-modified SELDI tiles are in different varieties, as substrates using hydrophobic interactions (e.g., C18, C4), hydrophilic, ionic, atomic (e.g., thiol), and antigen-antibody interactions that interact with proteins, nucleic acids, or other molecules with strictly defined physicochemical properties [86] (Figure 3).

SELDI is a technique that allows for rapid identification of various cancer biomarkers (e.g., prostate [87], pancreas [88], epithelial ovarian cancer [89], melanoma [90]) in complex biological matrices, such as saliva [91], serum [92], urine [93], or cerebrospinal fluid [94]. Furthermore, Yong Li et al. applied SELDI for searching for biomarkers of prostate cancer in urine and tear matrix [95]. Also in the case of Alzheimer’s disease, SELDI was used to analyze amyloid beta peptides in patients with different stage of dementia [96]. In addition, an attempt was made to look for biomarkers of Alzheimer’s disease through the use of SELDI [97,98]. The predominance of SELDI over the ELISA in this research results from the simultaneous identification of multiple peptides with MS in one measurement instead of a series of separate tests for each of the peptides. On the other hand, it was reported that the critical opinions about limitation of this technique result from its poor reproducibility, which limits its use in routine clinical diagnostics [99].

NALDI is a fast-growing and prospective field for applications for different analytical, environmental, and medical purposes, but also has its challenges and limitations. One of the limitations of this field is preparation of the target with deposited nanomaterials to get reproducible and accurate results, as nanomaterials can be labile in solid state after drying on the target. Large number of nanomaterials are used nowadays for this purpose: metal and metal oxide nanoparticles, carbon-based nanomaterials, nanocomposites, metal organic frameworks (MOFs). Gold nanoparticles attracted much attention because of their intrinsic advantages, such as large surface area, strong absorption efficiency in UV-Vis region, high chemical stability, easy modification, and preparation [100]. However, targets for NALDI analysis based on gold nanoparticles are mostly prepared through their in situ synthesis (directly before analysis). Solvents and precursor salts (silver nitrate, chloroauric acid) consumption is quite large in this case and the reaction takes, in general, more than 3 days (84 h) [101]. Therefore, the main challenge of NALDI is searching for a simple and effective method for preparation of NALDI targets cheaper than with the existing analogues based on chemically synthesized metal and metal oxide nanoparticles for detection of naturally-occurring low molecular weight compounds with low detection limits. 

Analysis of natural compounds by NALDI technique without the need for separation and without complex sample preparation can offer a fast and cheap method for their screening in plant materials and biological samples. For example, lipids and their distribution in human physiological liquids (sweat, blood, urine) and tissues give important information about biomarkers of different diseases. Techniques of NALDI imaging are being developed and employ conductive glass slides, as well as commercial targets (Bruker Daltonics, Germany), for tissue and tumor imaging [102]. Moreover, some important biomarkers can have quite homogeneous distribution in the tumor (tissue), but others have heterogeneous distribution, and despite this, NALDI technique is able to detect this heterogeneity [103]. Analysis of lipid species distribution by LC is complicated because of the need of their extraction from matrices and subsequent potential loss of biomarkers. However, LC-MS application for analysis of lipid species gives an advantage of quantitative analysis. Even though MALDI-technique has always been applied for semi-quantitative analysis owing to poor shot-to-shot and batch-to-batch reproducibility, this limitation is overcome by using internal standard calibration. Usually, isotopically labelled internal standards are expensive and not available, but cheaper internal standards with similar structures can also be used for quantification by MALDI approach. Application of NALDI target avoids the problem of poor shot-to-shot and batch-to-batch reproducibility, as the co-crystallization process that usually produces inhomogeneous mixtures and results in "hot spots" with conventional organic matrices does not take place [104]. It offers possibility for LMW quantification that is an important prospect for different kinds of metabolomics studies, as nanomaterials applied for NALDI targeting can serve not only for assisting ionization, but also for analyte clean-up in complex samples [105]. Another emerging application for developed targets is its application in the forensic field as well: latent fingerprints (LFP) are analyzed by the target to study the presence of organic molecules that can give "chemical information" about fingerprint composition. Chemical information from the fingerprint includes endogenous and exogenous compounds, such as drugs, explosives, toxins, and poisons. Endogenous compounds found on the skin are lipids, peptides, amino acids, proteins, urea, inorganic, and organic salts [101]. This information is used in forensic (for biometric identification and drugs consumption evaluation) and anti-doping analysis. NALDI target is a tool for LFP imaging by using TOF/TOF-MS analysis and microscopic equipment for creation of high-resolution optical images of the fingerprint, and immediately sent to the crime lab for rapid visual and computer-aided analysis [106]. In addition, the advantage of this target application in forensics for identifying drug consumption suspects and anti-doping analysis is confidence of the identity of the sample, as the fingerprint is unique for each person as also physical information is obtained by touching the surface with a finger. Moreover, determination of C18 steroids by GC-MS and GC-TOF-MS is complicated because of their high bioactivity and sticking to non-deactivated surfaces (some types of plastics and glass, GC column) resulting in potential analyte losses and low accuracy of results. Therefore, their analysis by NALDI is promising from an analytical point of view, owing to a simple sample preparation step prior to analysis and direct sample measurement without analyte separation in the column, thus causing analyte losses. Previously, various metal oxide nanoparticles (ZnO, Fe_2_O_3_, CeO_2_, TiO_2_) were synthesized and dusted directly on fingerprint surfaces spiked with drugs traces and without them, using a brush for detection of LMW drugs. Nanoparticles of zinc oxide were synthesized by microemulsions approach, however Fe_2_O_3_ nanoparticle application led to the best ionization performance [106]. 

Summary of the research papers with application of metal and metal oxide nanoparticles for analysis of LMW compounds in different matrices is presented in Table 2. Gold nanoparticles were synthesized in situ on a target plate (AuNPET target) for analysis of drugs and endogenous compounds in an onion bulb and chicken liver, as well as for fingerprints [101], amino acids, nucleosides, saccharides, and nucleic bases [107]. As mentioned before, the reaction took 84 hours, S/N ratio for serine and histidine was compared to S/N ratio obtained from ^109^AgNPET [108], and at 20 µg/mL of serine, the value accounted for 426 in the first case, and 259 for the silver target plate. Histidine adduct ratio (20 µg/mL) was equal to 510 with application of AuNPET and 104 for AgNPET, while uracil adduct showed the ratio of 599 for silver and 318 on AuNPET (10 µg/mL). Satisfactory S/N ratios were obtained with the use of ^109^AgNPET for almost all amino acids, except arginine (10). Later on, it was applied for analysis of amino acids distribution in blood samples [109]; spiking of samples with amino acids showed no suppression effect of the sample matrix. Preparation of the target is not as time-consuming as in the case of AuNPET target, but the cost is still relatively high. 

Another interesting approach of target preparation is vapor deposition of platinum [110], which is a good alternative for solvent-free homogeneous deposition, as the use of solvents for colloidal dispersions requires spray deposition and further drying on the target, which results in inhomogeneous distribution, and subsequently, poor reproducibility. The reported method allowed for identification of LMW compounds as saccharides, pigments, and drugs separated by TLC directly in ink jet printed on paper without extraction or concentration. A similar approach was applied before by the same research group for gold nanoparticles and identification of endogenous compounds in latent fingerprints. The complementary nature of the double images of LFP, such as optical and physical imaging, provides a physical pattern for personal identification, as well as chemical information from fingerprints for forensic investigations. To the best of our knowledge, endogenous compounds from fingerprints give important information on the person’s identity, for example, lipids distribution can be used not only as diseases biomarkers, but also for identification of age and sex of the person. The reported method allowed for successful identification of such compounds as verapamil (a calcium channel blocker for treatment of cardiovascular disease), or lipids that are commonly found in LFPs. In addition, authors demonstrated the advantage of breaking of the optical image into molecular images, which gives the possibility to separate two overlapping LFPs [111]. A magnetron sputtering system was also applied for deposition of gold thin film to commercial ITO conductive glass for MALDI-imaging for peptides analysis [112]. Spraying of colloidal silver on the target surface allowed for identification of wax compounds directly from plant leaves and flowers by attaching them with conductive double-sided tape. LMW compounds were registered as ^107^Ag and ^109^Ag adducts that proved the utility of spraying of colloidal silver for preparation of the target. Moreover, it allowed for evaluation of the distribution of wax compounds, as leaves and flowers did not undergo any sample pre-treatment, which also reduces time and costs of analysis, but requires the use of a computer-controlled syringe-pump [113]. 

Beside innovative methods of nanoparticles deposition, Au and Ag colloids were also deposited onto the target surface. Sacks et al. [114] deposited nanoparticles, pre-mixing them with analyte standards in different ratios, and drying them prior to analysis. They compared ionization trends with DHB matrix, as well as differently sized (2- and 5-nm) Au nanoparticles. Probably, such sample preparation allowed for registration of the analyte peaks, due to the high chemical stability of Au. However, the ionization mechanism is still not so clear, and there is no confidence if it will be applicable to real samples due to the biological matrix suppression effect. Interestingly, for diphenhydramine, multiple peaks were observed—protonated molecule, sodium, and potassium and Au [M-Au]^+^ adduct. Moreover, fragmentation of the molecule was observed, which suggests multiple zones of ionization, whereby desorption/ionization of an intact molecule and interaction of analyte closer to AuNPs possibly generates high-energy electrons and radical species. Authors provided quite insightful discussion of the observations with proposed structures of molecule fragments. Deposition of nanoparticles directly to the plate was performed with the following procedure: preparation of samples in sodium chloride solution, 1 µL deposition on the plate, drying in air, and deposition of 1 µL 13 nM of Au nanoparticles to the first layer and allowing them to dry. Ionization of analytes was compared with DHB matrix. It was concluded that AuNPs have high salt tolerance; the increase of salt concentration did not affect ionization and showed suppression effect only at 500 mM of NaCl. This procedure was also applied for urine samples without any pre-treatment; it allowed for registration of peaks of urea, creatinine, uric acid, and glucose, while DHB matrix showed suppression effects. As normal urine samples contain 130 nM of NaCl, it did not prevent registration of mass spectra of urine samples with AuNPs application. However, authors stated that the method was still not sensitive for neutral steroids and probably its coupling with extraction from urine samples would provide better results [115]. Zinc oxide nanoparticles prepared by suspension in methanol and irradiated by ultrasonic agitation for 2 h were applied for LDI-MS, also by deposition and drying on the target surface. It is important to note that they were successfully applied without liquid matrix or buffers, such as citric acids, with a low background noise. Shot-to-shot reproducibility of testosterone signals was examined and RSD value for 50 different sample spots was 27%, as compared to 81% for DHB matrix. The sweet spot was better overcome by ZnO nanoparticles, confirming homogeneous distribution and suggesting the possibility for quantification [116]. 

An improved DIOS method was developed by electroless plating of silver nanoparticles on porous silicon. Addition of 4-aminothiophenol slowed down the plating speed and self-assembled monolayers were formed, thus enhancing stability, sensitivity, and reproducibility. Storage in air for several weeks to a month also did not affect the reproducibility [117]. Even though the method was proved to be efficient, there is still a need to reduce the costs of analysis and simplification of the target preparation procedure.

In summary, MALDI and NALDI are two ionization methods that complement each other. They allow for the analysis of ionized biologically active compounds in a wide range of molecular weights. MALDI is successfully used in the analysis of macromolecules—mostly proteins, but also lipids, cyclitols, or antibiotics. Analysis of low molecular weight compounds, especially small drug molecules, pigments, or amino-acids, is effective in the NALDI system. Complementarity between MALDI and NALDI creates new interpretation possibilities. This technique has found a wide application in bioanalytics and will certainly be further developed due to its high potential.

## Figures and Tables

**Table 1:** Short-review of matrix-assisted laser desorption/ionization with time-of-flight mass spectrometry (MALDI-TOF MS) methods used for the identification and analysis of different proteins.

Protein	Molecular Mass [kDa]	Sequence Coverage (SC)	Post-Translation Modifications	Reference
Connexin26	-	71.3%	acetylation, hydroxylation, γ-carboxyglutamation, methylation, phosphorylation	[28]
Carbonic anhydrase IX (CA IX)	44.48	-	-	[40]
α_s1_-CN	23.61	75%	phosphorylation	[1]
	25.0	54%		[45]
β-CN	23.99	37–49%		[1]
	29.0	51%		[45]
κ-CN	19.0	33–35%	phosphorylation	[1]
	-	-	O-glycosylation	[37]
α-lactalbumin	14.199	-	-	[31]
	-	-	N-glycosylation	[29]
β-lactoglobulin	18.397	-	-	[32]
	-	-	N-glycosylation	[29]
Lactoferrin	77.167-81.189	19–25%	Glycosylation	[34]
	84.011	-		[35]
Bovine serum albumin (BSA)	62.0	35.7–43.9%	-	[8]
Transferrin	-	41.8–56.4%	-	[9]
	-	-	N-glycosylation	[39]
	79.562	-	O- and N-glycosylation Acetylation	[46]

**Table 2:** Review of application of metal and metal oxide nanoparticles for laser desorption/ionization (LDI) detection.

Matrix	Analytes, Sample	Synthesis	Analytes Deposition	LOD (Limit of Detection)	Reference
Gold nanoparticles, target plate (AuNPET)	Pentedrone, diphenylamine, metronidazole and endogenous compound (saccharides, ionic and non-ionic glycerides, amino acids, fatty acids, sulfides, sulfoxides, phenols) in human FP, onion bulb, chicken liver	In situ on target plate (84 h reaction)	A volume of 0.5 µL of extract of liver, 1 µL of onion extract, was placed directly on AuNPET, air dried, and measured within 60–1000 *m/z* range	n/d (non-detected)	[101]
AuNPET	Nucleosides, saccharides, amino acids, glycosides, nucleic bases	In situ	Stock solution (1 mg/mL) of each analyte was prepared, diluted, and 0.3 µL of the final solution was applied to the AuNPET and air- dried	n/d	[107]
Gold nanoparticles (AuNPs) (2 and 5 nm)	Carbohydrates, steroids, bile acids	Ready to use from manufacturer	Analytes were dissolved into 3:1 acetonitrile: water with 0.1% formic acid (FA) at a 2.0 × 10^−2^ M and diluted to make matrix-to-analyte ratios of 1 × 10^4^:1, 1 × 10^5^:1, and 1 × 10^6^, 3 μL of each solution was spotted and dried on separate wells of a stainless steel plate	n/d	[114]
AgNPET	Amino acids (AAs) from blood samples	In situ from silver-109 trifluoroacetate dissolved in tetrahydrofuran (THF)	Volumes of 0.5 μL of amino acid solutions diluted 10 times were placed directly on target plate and air dried, target was inserted into MS apparatus; 1 µL of plasma was dissolved in 999 μL of ultrapure water and used in this diluted form; plasma was spiked with 515 ng of Ser or 38 ng of Phe	LOD for AAs (pg/mm^2^): Arg—0.9; His—0.13; Ile—0.06; Met—0.13; Ser—0.16; Phe—0.1; Tyr—2.6; Ala—64 (fg/mm^2^); Asp—12; Cys—41; Lys—2.3 pg/spot	[109]
AgNPET (monoisotopic ^109^Ag)	AA: Trp, His, Ser, Met, Arg, Pro, Cys, Gln, Glu, Asp, Ala, Tyr, Leu, and Phe	Silver trifluoroacetate (200 mg) was dissolved in anhydrous, inhibitor-free THF (250 mL) and the solution poured into a large beaker containing a target plate. Solid 2,5-dihydroxy acid benzoic (DHB) (400 mg) was added and, following stirring, the solution was left for 24 h	Samples (0.5 μL) of the final solutions (dissolved in water) were applied to the sample plate and air-dried	Signal-to-noise (S/N) ratio: Gln, Ala, Phe, Leu, Glu, Tyr, Cys, Ser higher than 200; Arg—10.	[110]
Silver nanoparticles (AgNPs)	Different lipid classes from mouse and rat tissues, including brain, kidney, liver, and testis	Silver layers were deposited on top of the tissue sections using a sputter coater	All tissues were sectioned at 14 μm thickness using a cryostat and thaw-mounted on ITO coated slides; tissues were dried in desiccators prior to the silver deposition	n/d	[118]
AgNPs	triglycerides (TAG, C8−C16) and phosphatidylcholines (PC): 1,2-dimyristoyl-sn-glycero-3-PC (DMPC), 1,2-dipalmitoyl-sn-glycero-3-PC (DPPC), and 1-palmitoyl-2-oleoyl-sn-glycero-3-PC (POPC)	Porous AgNPs-impregnated thin films were prepared by the sol-gel method	A mixture of analytes was spotted directly to the surface and analyzed	n/d	[119]
AgNPs	Tetrapyridinporphyrin (TPyP), oligomers of polyethylene glycol, peptide of oxytocin	Electroless plating of nanoparticles on porous silicon for desorption–ionization on porous silicon (DIOS)	PEG was dissolved in acetonitrile (ACN), TPyP in dichloromethane (DCM), and oxytocin in MILLIQ water, diluted and 1 µL was dropped onto the silver film of the substrate, air-dried, and chip was introduced immediately into the mass spectrometer	LODs, fmol: TPyP—1.3 Oxytocin—1.5	[117]
Silver colloids	Cuticular wax metabolites from *Arabidopsis thaliana* leaves and flowers	Commercial colloidal silver was sprayed to the target; the parameters for spraying were optimized; the sample was air-dried after each application	Collected flower and leaf samples from plants were stored in the ice before attaching them to sample plates; they were attached onto a stainless steel target plate of similar dimensions as a microscope glass slide using a conductive double-sided tape	n/d	[113]
AgNPs/zinc oxide nanorods (ZnO NRs)	Amino acids, polyethylene glycol (PEG) (MW 2000), Rhodamine 6G (R6G)	ZnO NRs were fabricated through seed layer-assisted hydrothermal method; then ZnO NRs were modified with TPFS and decorated with evaporated Ag NPs; seed layers was formed on silicon wafers	A volume of 3 µL of analyte solution was added on substrate, then dried in air at room temperature; the substrate was mounted on a target plate using double-sided carbon tape	Arg—1.0 × 10^–15^ M; PEG—2000–1.0 × 10^–10^ M; R6G—1.0 × 10^–15^ M	[120]
Zinc oxide nanoparticles (ZnO NPs)	Small drug molecules (nortriptyline, amitriptyline, imipramine, promazine) in latent fingerprint	Nanoparticles were synthesized by microemulsions and dried at 110 °C overnight	The thumb was wiped across the forehead for 10 s and then pressed against the target plate or a glass slide for 10 s, leaving an impression on the surface; following deposition, NPs or the DHB organic matrix were applied to the LFP by dusting using a brush; fingerprint surface was spiked with 3 protocols	n/d; (relative standard deviations (RSDs) for [M-H]^+^ in %: nortriptyline—0.094; amitriptyline—0.202; imipramine—0.036; promazine—0.199	[108]
AuNPs	Peptide fragments from standard protein digests of bovine serum albumin, bovine catalase, and bovine lactoperoxidase	Gold thin film was deposited on indium tin oxide (ITO) -conductive glass	Protein digests were dissolved in 80% ACN and 20% citrate buffer solution (3:1 50 mM ammonium citrate/100 mM citric acid) and 0.2 µL of each digest were spotted on the hydrophilic etched gold spots	angiotensin I peptide—8 fmol	[112]
AuNPs	Testosterone, progesterone, cortisol, ribose, glucose, maltose, 5- 5-hydroxyindolacetic acid (HIAA), tryptophan, gangliozyd (GM1), angiotensin I from urine samples	A solution of the AuNPs was prepared by the chemical reduction of metal salt precursor in a liquid solution	Urine samples were directly deposited onto the sample plate and allowed to dry in air; then, an equal volume of 13 nM Au NPs or 20 mg/mL DHB was deposited onto the first layer and allowed to dry in air before MALDI—TOF-MS measurements	LOD, nm: Testosterone—188; Progesterone—389.8; Cortisol—641; Ribose—1395; Glucose—393.4; Maltose—785.3; 5-HIAA—46.5; Trp—141.5; GM1—1648.4; Angiotensin I—5115.7	[115]
ZnO NPs	Polyethylene glycol, polystyrene and polymethylmethacrylate, oligosaccharides, lipids	ZnO was suspended to achieve 10 wt % in methanol; the suspended solution was irradiated by ultrasonic agitation for 2 h	Each 0.6 µL of the ZnO dispersed solution and analyte solutions were placed on a stainless-steel sample target (384 wells) and dried at room temperature; NaI (0.1 mM) was added to all sample solutions as cationizing agent, except for verapamil hydrochloride	Β-cyclodextrin and hexa-N-acetylchitohexaose—1 pmol	[116]
Platinum nanoparticles (PtNPs)	Saccharides, pigments, and drugs	Vapor deposition Pt deposition on the target imaging sample was performed by commercially available magnetron sputtering device	Pt-deposited sample was mounted onto a holder plate and fixed using electrically conductive carbon tape	n/d	[110]
AuNPs	Endogenous chemicals in latent fingerprints (LFPs)	Vapor deposition by sputtering	To prepare sebum-rich LFPs, the donor wiped his thumb on his forehead for about 10 s, and then pressed his thumb on the desired substrates gently for about 10 s	n/d	[111]
